# Exploring the Effects of a Yoga Intervention on Stress and Coping Self-Efficacy on People Living With HIV: A Randomized Controlled Trial

**DOI:** 10.1097/jnr.0000000000000719

**Published:** 2026-01-12

**Authors:** Jufri HIDAYAT, Miao-Yen CHEN, Chieh-Yu LIU, Wen-I LIU, Kuei-Min CHEN, Piao-Yi CHIOU, Stefani PFEIFFER

**Affiliations:** 1 Department of Nursing, School of Nursing, National Taipei University of Nursing and Health Sciences, Taipei, Taiwan; 2 Department of Health Care Management, National Taipei University of Nursing and Health Sciences, Taipei, Taiwan; 3 School of Nursing, College of Nursing, Kaohsiung Medical University, Kaohsiung, Taiwan; 4 School of Nursing, College of Medicine, National Taiwan University, Taipei, Taiwan; 5 International Center, National Taipei University of Nursing and Health Sciences, Taipei, Taiwan

**Keywords:** HIV, AIDS, stress, coping self-efficacy, yoga

## Abstract

**Background:**

Human immunodeficiency virus (HIV) remains a serious challenge to public health. Stress is a primary issue affecting HIV care because it is highly prevalent among people living with HIV and negatively affects quality of life in this population.

**Purpose:**

This study was designed to examine the efficacy of a yoga intervention in reducing stress and enhancing coping self-efficacy in people with HIV.

**Methods:**

A parallel-group, randomized controlled trial with single blinding and repeated measures was used. The intervention group engaged at home in eight biweekly online Hatha yoga classes of 120 min (two 60-min sessions) in length. The effects were assessed at baseline, at the end of the 2-month intervention, and at 1 month after the end of the intervention.

**Results:**

Sixty-six people were enrolled as participants, five of whom were lost to follow-up at the second assessment. Sixty-one participants took part in the third assessment. After practicing yoga for 8 weeks, the intervention group had lower mean stress scores and higher mean coping self-efficacy scores than the control group.

**Conclusions/Implications for Practice:**

The yoga intervention applied in this study was shown to effectively reduce perceived stress and strengthen coping self-efficacy in patients with HIV. This study adds evidence gathered in a new social context (Bali, Indonesia) to existing research showing practicing yoga to be effective in reducing stress in patients with HIV. Yoga is a promising complementary intervention that may be offered to patients with HIV suffering from stress.

## Introduction

Human immunodeficiency virus (HIV) remains a public health challenge four decades after it emerged as a global epidemic. Indonesia, one of the largest and most populous countries in the world, is also among the hardest hit by the HIV epidemic. Stress has been documented as one of the most frequently diagnosed mental health issues occurring among people living with HIV (PLWH; Liu et al., 2023). Most patients experience stress at multiple points along their HIV treatment continuum. For example, stress occurs when someone is first diagnosed with HIV, when starting antiretroviral treatment, while remaining under care, or when faced with challenges to medication adherence (Liu et al., 2023; Swinkels et al., 2024). Stress remains a primary issue in HIV care because it is highly prevalent among PLWH and affects quality of life in this population negatively. Stress in PLWH can often go unrecognized by health care professionals as well as by patients themselves (Henry-Okafor et al., 2022; Mbalinda et al., 2023). Moreover, when recognized, assessment and treatment are often not initiated and, even when they are, the treatment for stress may not be successful (Henry-Okafor et al., 2022).

### Background

Many researchers have reported global morbidity and mortality related to stress to be increasing dramatically (Liu et al., 2023; Stoner et al., 2023) and some have investigated the efficacy of nontraditional stress management as alternative treatments. Yoga is one of the complementary therapies increasingly used in nursing and other health care systems. Evidence highlights yoga as a promising modality for treating stress and maintaining the health and well-being of PLWH (Hoffmann et al., 2024; Jiang et al., 2021). However, its use in HIV populations in Indonesia remains limited. The definition of yoga in this study follows that of Yatham and colleagues, who defined yoga as “a holistic mind–body practice for physical and psychological health that combines numerous techniques to build strength and flexibility that could train attention, including physical posture and exercises, deep relaxation, and meditation or mindfulness techniques” (Yatham et al., 2023, pp. 3–6).

Stress management interventions such as yoga may be taught both in traditional face-to-face classes and virtually. Although people nowadays have many choices and free access to online yoga videos, patients in need of treatment are advised to seek assistance from professional yoga teachers or health care professionals. Virtual yoga interventions provide an efficient, alternative approach that offers an evidence-based practice for reducing the negative consequences of stress (Miyoshi et al., 2022). A recent randomized controlled trial (RCT) conducted in New York to investigate the effect of tele-yoga on stress management efficacy during the COVID-19 pandemic found 4 weeks of tele-yoga to be safe and feasible and to have significantly improved well-being and reduced stress in the participants (Trevino et al., 2021). Many nursing interventions may be implemented online. Thus, shifting yoga from a traditional face-to-face practice to a virtual format is of potentially great value in terms of adapting established therapies to changing technologies and lifestyles.

The use of alternative treatments such as yoga as health care interventions has grown in recent years (Ng et al., 2023). However, no gold standards or treatment guidelines currently exist for managing patients with HIV with stress-related symptoms. Most often, pharmacological treatments are the first choice for managing stress. Considering that many patients with HIV suffer from stress-related symptoms, there is an urgent need for researchers to investigate complementary and alternative stress management techniques, such as yoga, further.

Evidence has shown that yoga improves the coping self-efficacy of patients in managing their disease. Baghbani et al. (2023) showed that high levels of coping self-efficacy facilitate the development of adaptive coping strategies, which, over time, may make individuals less susceptible to stress and better able to use constructive coping mechanisms when problems arise. For example, they may reduce their engagement in cognitive escape and avoidance activities and other maladaptive coping behaviors. Previous research has shown that yoga provides an alternative focus to negative or self-defeating thoughts and has a positive impact on thought content by increasing feelings of self-efficacy and confidence (Baghbani et al., 2023). Similarly, Marshall et al. (2022) observed that the level of self-efficacy in yoga participants to increase after completion of a yoga trial and those engaging in yoga as a self-care behavioral treatment to be more likely to experience increases in self-efficacy and self-motivation.

The potential of yoga and its benefits remain understudied in patients with HIV. Although yoga is increasingly being used as an adjunct to clinical practice, and researchers are particularly interested in its benefits for patients with stress-related syndromes, information on the effects of yoga on patients who are HIV-positive, in particular, remains limited. Most previous studies on the advantages of yoga interventions did not include PLWH. Therefore, the primary objective of this study was to explore the efficacy of yoga in reducing stress. The secondary objective was to improve patient self-efficacy. The research question was phrased as follows: “Does participation in an eight-week online yoga intervention reduce stress and improve self-efficacy in PLWH in Indonesia to levels significantly better than in their peers receiving usual care?”

## Methods

### Design

A parallel group RCT design with single blinding and repeated measures was used in this study. The participants were assigned to either the intervention (yoga) or control (routine care) group. The independent variable was the yoga intervention, and the dependent variable was stress. In addition to assessing stress, a demographic characteristics datasheet, health status survey, Perceived Stress Scale (PSS), and Coping Self-efficacy Scale were used in data collection. The mean differences between variable scores at baseline and after intervention completion were examined.

### Participants and Setting

This study was conducted online using the Zoom video call application, and the participants practiced the yoga intervention at home. The researcher and case manager, who both work in a health care center, collaborated on recruiting participants from Jumpandang Baru’s primary health care center in Makassar City, Indonesia. The recruitment process started when patients attending the clinic for their monthly follow-up and medication administration were invited to participate in the study. Also, the case manager called patients by telephone and informed them about the study, and if they agreed to participate, they were then invited to come to the clinic, where the nurses and researcher further explained the study. Those patients who agreed to participate signed written informed consent. Only patients who agreed to participate were included in this study.

The inclusion criteria were (1) a diagnosis of HIV; (2) newly diagnosed or having taken antiretroviral medication for at least 1 month; (3) at least 18 years old; (4) had regular access to a desktop, laptop, or smartphone; (5) had reliable internet access; (6) fluent in Bahasa Indonesia; and (7) willing to participate and sign the consent form. The exclusion criteria were (1) performing yoga or another mind–body exercise for at least 1 month before the start of this study; (2) not in physically fit condition; (3) diagnosed with musculoskeletal problems, for example, osteoporosis, osteoarthritis, bone injury, and spine problems; (4) having difficulty maintaining balance; and (5) currently pregnant or planning to become pregnant during the study period.

### Intervention

In this study, the researcher provided the intervention group with 2 months of Hatha yoga classes for stress management. The researcher chose Hatha yoga over other types of yoga due to its focus on relaxation, mindfulness, and stress reduction and its allowing participants to move slowly and carefully into various positions that test bodily strength and flexibility. All of the participants in the intervention group were required to complete 120 min of yoga per week (60 min per session) following the home-based, online yoga sessions for 8 weeks with a professionally certified yoga instructor who had prior experience teaching yoga to PLWH. The intervention was delivered by the yoga instructor using a concurrent online course, which was livestreamed. No prerecorded video was provided. Study data were collected at three time points, including baseline (T_1_; before the yoga intervention began), immediately after completion of the 2-month intervention (T_2_), and 1 month after completion of the intervention (T_3_), to evaluate the longitudinal effects of yoga on stress. A week before commencing the intervention, an online face-to-face meeting was arranged for all of the participants in the intervention group to explain how to prepare for the intervention.

To improve intervention fidelity and treatment adherence, the researcher and yoga instructor worked together to monitor the participants during the yoga sessions. The researcher developed a yoga protocol aimed at ensuring participants performed the yoga body poses appropriately. The yoga intervention procedures followed those described in Wimberly et al. (2018), which targeted a population similar to that in this study and reported significant results (*p*<.05). Anticipating the risk of injury during the study, the researcher and the clinic made sure an ambulance was on call and available to pick up a participant injured during an intervention session from their home and that support and treatment would be provided at no cost to them. The intervention group received reminders by telephone 1 day in advance to attend the weekly yoga sessions. In this study, the participation rate in the yoga intervention was ~93%.

### Control Group

In this study, the control group received standard care consisting of programs regularly provided by Jumpandang Baru Clinic, including a general health education program and physical check-ups. Both the control and yoga groups received an oral antiretroviral regimen. The control and yoga groups included 33 participants each.

### Ethics Statement

The study proposal and instruments were submitted to two institutional review boards at, respectively, the Makassar Health Department (No: 440/306/PSDK/VII/2022) and South Sulawesi Province (No: 070/1644/-II/BKBP/VII/2022). In addition, this trial was registered with Clinicaltrials.gov (RCT registration number: NCT05503680).

### Primary Outcomes

The primary outcome of this study was perceived stress. The 10-item Perceived Stress Scale (PSS) was used to measure stress in this study. The items assess on a scale ranging from 1 to 10 the frequency of feelings and thoughts related to events and circumstances occurring during the previous month (Cohen et al., 1983). The 10-item PSS has demonstrated good internal consistency and validity across multiple studies. In particular, its Cronbach’s α and test–retest reliability range from .78 to .91 (Cohen et al., 1983). Higher PSS scores are associated with higher levels of psychological stress (Cohen & Janicki-Deverts, 2012). This well-known scale has been translated into Indonesian and validated psychometrically for midwives by Indonesian scholars Purnami et al. (2019), who reported a Cronbach’s α of >.70.

### Secondary Outcomes

Coping self-efficacy, the secondary outcome of this study, was measured using the Coping Self-Efficacy Scale developed by Chesney et al. (2006). This 26-item scale is used to assess confidence in performing coping activities when confronted with life obstacles. This scale has been translated into Indonesian and validated by Sarry and colleagues with good reliability (α=.96; Sarry et al., 2021).

### Sample Size

The minimum sample size for this study was determined with power analysis using four components: degree of significance or alpha (α), sample size, population effect size, and power (1−β err prob). The sample size was calculated based on a known effect size of the primary outcome variable (stress management) from a previous study that used a yoga intervention to measure stress in a similar population with an effect size of 0.21 (Wimberly et al., 2018). G-Power 3.1.2 computer software was used for the calculation, with power set at 0.90, β=0.1, and α set at 5%. A two-tailed test was designed. To attain an effect size of 0.21 between the intervention and control groups, the total sample required was 50. Adding a 30% loss-to-follow-up and low-yoga-attendance buffer increased this number to a target sample size of 66, with 33 in each group.

### Randomization, Sequence Allocation, and Concealment

Participants in this study were randomized using the internet website “Randomizer.org” (https://www.randomizer.org). The researcher prepared sealed envelopes for allocation to ensure participant group assignments were not known in advance. Once participants had given their informed consent, the envelopes were opened, and the participants were assigned to either the intervention group or the control group. During the study, only the researcher was aware of individual group allocations. Moreover, the researcher confirmed that the participants did not know who was allocated to which group before completion of the study.

In this study, all patients with HIV undergoing antiretroviral treatment in Jumpandang Baru health care center, Tello District, Makassar City, Indonesia, were recruited for potential inclusion. According to the clinic, the total HIV-positive population taking ART treatment was 659. This population was vetted based on the eligibility criteria included in the CONSORT (Consolidated Standards of Reporting Trials) diagram. The first assessment (T_1_) was conducted to set the preintervention baseline; the second assessment (T_2_) was implemented upon the conclusion of the 2-month intervention, and the third assessment (T_3_) was implemented 1 month postintervention to evaluate the longer-term effects of yoga on stress reduction in the sample. Five hundred and ninety-six patients were excluded due to not meeting the inclusion criteria (*n*=356), declining to participate (*n*=198), scheduling conflicts (*n*=29), or lack of interest in the yoga intervention (*n*=10). The 66 enrolled participants were then randomized using the aforementioned internet website, with 33 allocated to the intervention group and 33 to the control group. Baseline data were acquired from all of the participants at T_1_. T_2_ data were collected immediately after completion of the 2-month of yoga intervention, with two in the intervention group lost to follow-up due to moving to another city (*n*=1) and scheduled surgery (*n*=1) and three in the control group lost to follow-up due to lost contact (*n*=1), passed away due to heart attack (*n*=1), and moving to another city (*n*=1). T_3_ data collection was performed 1 month after completion of the yoga intervention, with no additional participants lost to follow-up, leaving data from 31 participants in the intervention group and 30 participants in the control group available for data analysis.

To minimize bias, the approach used in this study followed the CONSORT flow diagram for RCTs evaluating interventions. The CONSORT item checklist was used by the researcher to report study findings. During study implementation, the researcher worked to retain the participation of all participants via telephone calls and follow-up care, resulting in a low attrition rate. Moreover, the reasons for participants’ withdrawal from this study do not relate directly to the intervention. The completion rate of this intervention study was 93.9% for the Per-Protocol Analysis (PP). The control group is 90.9% for the PP.

### Blinding

A single-blind randomized controlled trial was chosen as the most appropriate method to test the effectiveness of the treatment in this study. Single blinding applies to the data collector, who may otherwise bias results through their awareness of the allocations. The blinding of data collectors and outcome adjudicators is crucial to ensuring the unbiased ascertainment of outcomes. In addition to blinding, to prevent contamination between the intervention and control groups, the researcher asked the intervention group and the health care staff at the clinic not to reveal the intervention to other patients with HIV.

### Data Collection

Participant recruitment began after permission was granted. The case manager invited all patients who were HIV-positive and patients who visited the clinic for routine monthly medication refills to come to the clinic to learn about the study. Data were collected between August and September 2022 by a research assistant. The researcher and data collector worked together to ensure data accuracy. The participants, doctors, case managers, and the chief of the clinic all helped to facilitate and accomplish the study objectives.

### Statistical Analysis

Before performing statistical procedures, all data were entered into a computer and checked for errors. The data were analyzed using SPSS software version 20.0 (IBM Corp., Armonk, NY, USA). Data for all of the variables were manually coded before analysis. Statistical significance was defined as an alpha value of α=.05. Also, demographic characteristics were described using descriptive statistics, with frequencies and percentages obtained for categorical variables and means and standard deviations obtained for continuous variables. The data for independent and dependent variables were tested to determine their distributions and normality using plot histograms, logs, and square roots and by checking residuals. The generalized estimating equation (GEE) technique was used to model each result and determine associations between variables while accounting for correlated data within repeated measures (Hardin & Hilbe, 2007).

## Results

**Figure 1:**
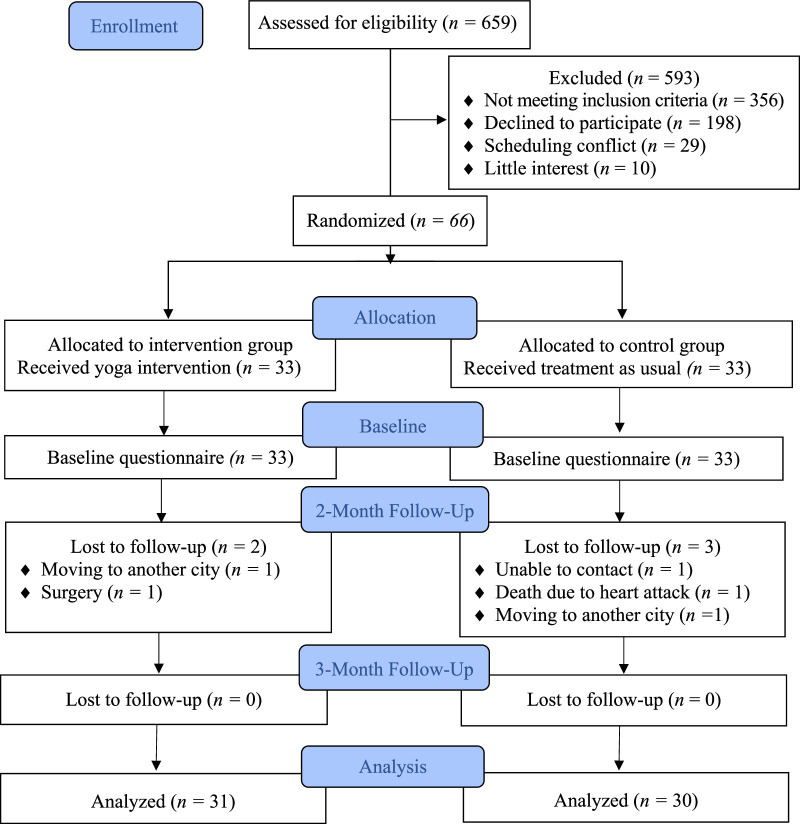
Consolidated Standards of Reporting Trials (CONSORT) Diagram

A total of 659 individuals were assessed for eligibility, with 356 not meeting the inclusion criteria and 198, 29, and 10 declining to participate due to, respectively, privacy concerns, scheduling conflicts, and lack of interest. The remaining 66 PLWH agreed to participate and signed the informed consent letter, with 33 allocated to the control and intervention groups, respectively. Thirty-one and 30 participants in the intervention and control groups, respectively, provided valid data at all three time points. The participant selection process used in this study is described in Figure 1.

### Patient Characteristics

As shown in Table 1, most intervention and control group participants were male (*n*=23 [74.2%] and *n*=20 [66.7%], respectively), single (*n*=24 [77.4%] and *n*=22 [73.3%], respectively), 26–27 years old, and self-identified as homosexual (*n*=16 [51.6%] and *n*=16 [53.3%], respectively). Although the majority of the participants were open about their sexual preferences, some refused to answer questions related to this aspect (*n*=9 [29%] and *n*=5 [16.7%], respectively). With the exception of duration of HIV infection (*p<*.01), no significant between-group differences in terms of demographic characteristics were found.

**Table 1:** Participant Demographics

Variable	Intervention Group (*n*=31)	Control Group (*n*=30)	*t/*χ^2^	*p*
	*n* (%)	*n* (%)		
Age (years, *M*±*SD*)	27.23±6.15	26.13±3.88	*t* = −0.827	.06
Gender			1.127	.57
Male	23 (74.2)	20 (66.7)		
Female	6 (19.4)	9 (30.0)		
Transgender	2 (6.5)	1 (3.3)		
Sexual orientation			1.727	.42
Heterosexual (straight)	6 (19.4)	9 (30.0)		
Homosexual	16 (51.6)	16 (53.3)		
Bisexual	0 (0.0)	0 (0.0)		
Prefer not to answer	9 (29.0)	5 (16.7)		
Educational level			0.553	.46
Elementary school	0 (0.0)	0 (0.0)		
Secondary school	20 (64.5)	22 (73.3)		
Undergraduate	11 (35.5)	8 (26.7)		
Occupation			2.640	.62
Housewife	2 (6.5)	4 (13.3)		
Student	8 (25.8)	5 (16.7)		
Employed	12 (38.7)	14 (46.7)		
Civil servant	3 (9.7)	4 (13.3)		
Entrepreneur	6 (19.4)	3 (10.0)		
Marital status			0.771	.86
Single	24 (77.4)	22 (73.3)		
Married	3 (9.7)	2 (6.7)		
Divorced	3 (9.7)	5 (16.7)		
Widowed	1 (3.2)	1 (3.3)		
Monthly income ^a^			2.499	.48
<Rp 1,000,000 (USD 65)	3 (9.7)	6 (20.0)		
Rp 1,000,000–3,000,000 (USD 65–193)	10 (32.3)	7 (23.3)		
Rp 3,000,001–5,000,000 (USD 193–321)	11 (35.5)	13 (43.3)		
Rp 5,000,001– 10,000,000 (USD 321– 642)	7 (22.6)	4 (13.3)		
Living status			1.105	.58
Living alone	15 (48.4)	18 (60.0)		
With family	12 (38.7)	10 (33.3)		
With friends	4 (12.9)	2 (6.7)		
Comorbidities			0.317	.57
None	29 (93.5)	29 (96.7)		
Yes	2 (6.5)	1 (3.3)		
Social support			2.156	.54
None	2 (6.5)	1 (3.3)		
Families	9 (29.0)	14 (46.7)		
Friends	10 (32.3)	8 (26.7)		
Peers	10 (32.3)	7 (23.3)		
Length of time (years) since diagnosis of HIV	1.37±1.12	0.94±0.57	*t* = −1.917	< .01

*Note.* Rp = Indonesian Rupiah.

^a^ Exchange rate: 1 USD=Rp15,593 on December 25, 2022.

### Within-Group Comparison

The within-group comparisons for the three different time measurements: baseline, week 8, and week 12 are shown in Table 2. The intervention group reported a significantly lower mean level of stress at T_2_ (i.e., 20.81 [*SD*=4.22] at baseline vs. 6.97 [*SD*=2.37] at T_2_). However, the mean stress score increased between T_2_ and T_3_ (i.e., to 13.48 [*SD* =1.34]). Similarly, a significant improvement in the intervention group was shown over time in the mean coping self-efficacy score (*p*<.01), which rose from 159.68 (*SD*=23.76) at baseline to 208.71 (*SD*=10.46) at T_2_ and then slightly decreased to 197.23 (*SD*=8.56) at T_3_.

**Table 2:** Within-Group Comparisons Over Time

Variable	Intervention Group (*M*±*SD*)				Control Group (*M*±*SD*)			
	Baseline	Week 8	Week 12	*p*	Baseline	Week 8	Week 12	*p*
Overall health status	2.35±1.31	1.65±0.48	2.84±2.83	< .01 ** T_2_ → T_1_=.03 T_3_ → T_1_=.49 T_3_ → T_2_=.00	3.10±0.96	2.83±1.10	2.83±1.10	.28
Systolic blood pressure (mm Hg)	118.39±9.34	117.74±5.60	121.2±20.61	.67	116.33±6.69	116.6±6.61	115. 67±6.26	.41
Diastolic blood pressure (mm Hg)	74.19±10.58	75.1±8.90	74.19±10.58	.51	72.00±8.05	72.33±8.17	71. 00±8.45	.31
Body mass index (kg/m^2^)	22.77±2.14	22.8±2.06	22.84±2.06	.16	21.83±2.03	21.93±2.01	22.94±2.01	.07
Smoking to cope with stress in the past month (%)	0.29±1.13	0.00±0.00	0.00±0.00	.14	0.40±1.25	0.50±1.33	0.50±1.33	.37
Perceived stress scale	20.81±4.22	6.97±2.37	13.48±1.34	< .01 ** T_2_ → T_1_=.00 T_3_ → T_1_=.00 T_3_ → T_2_=.00	21.63±3.77	24.33±3.44	22.40±2.03	< .01 ** T_2_ → T_1_=.00 T_3_ → T_1_=.49 T_3_ → T_2_=.00
Coping self-efficacy score	159.68±23.76	208.71±10.46	197.23±8.56	< .01 ^*^ T_2_ → T_1_=.00 T_3_ → T_1_=.00 T_3_ → T_2_=.00	184.33±21.76	178.57±19.14	181.43±16.72	< .01 ** T_2_ → T_1_=.00 T_3_ → T_1_=.06 T_3_ → T_2_=.00

*Note. p* value was obtained from repeated measures ANOVA. T_1_ = Time one (pretest), T_2_ = Time two (posttest one), T_3_ = Time three (posttest two).

*p<.05. **p<.01.

### Between-Group Comparison

Between-group comparisons of mean perceived stress and coping self-efficacy scores are given in Table 3. While mean perceived stress scores were similar in both groups at baseline (*p>*.05), between-group differences were significant at both T_2_ (24.33 [*SD*=3.44] vs. 6.97 [*SD*=2.37]) and T_3_ (13.48 [*SD*=1.34] vs. 22.40 [*SD*=2.03]). Similarly, while mean coping self-efficacy was similar in both groups at baseline (*p>*.05), between-group differences were significant at both T_2_ (208.71 [*SD*=10.46] vs. 178.57 [*SD*=19.14]) and T_3_ (197.23 [*SD*=8.56] vs. 181.43 [*SD*=16.72]).

**Table 3:** Between-Group Comparisons Over Time

Variable	Baseline (*M*±*SD*)			Week 8 (*M*±*SD*)			Week 12 (*M*±*SD*)		
	Intervention	Control	*p*	Intervention	Control	*p*	Intervention	Control	*p*
Overall health status	2.35±1.31	3.10±0.96	.01**	1.65±0.49	2.83±1.12	<.01**	2.84±1.10	2.83±1.12	.86
Systolic blood pressure (mm Hg)	118. 39±9.34	116.33±6.69	.26	117.74±5.60	116.67±6.61	.12	121.29±20.61	115.67±6.26	.10
Diastolic blood pressure (mm Hg)	74.19±10.58	72.00±8.05	.21	75.16±8.90	72.33±8.17	.95	74.19±10.58	71.00±8.45	.31
Body mass index (kg/m^2^)	22.77±2.14	21.83±2.03	.59	22.84±2.06	21.94±2.01	.53	22.84±2.06	21.94±2.01	.53
Smoking to cope with stress in the past month (%)	0.29±1.13	0.40±1.25	.49	0.00	0.50±1.33	<.01**	0.00	0.50±1.33	<.01**
Perceives stress score	20.81±4.22	21.63±3.77	.65	6.97±2.37	24.33±3.44	.01*	13.48±1.34	22.40±2.03	.03*
Coping self-efficacy score	159.68±23.76	184.33±21.76	.71	208.71±10.46	178.57±19.14	<.01**	197.23±8.56	181.43±16.72	<.01**

*Note. p* value was obtained from independent t tests. T_1_ = Time one (pretest), T_2_ = Time two (posttest one), T_3_ = Time three (posttest two).

*p<.05. **p<.01.

### GEE Analysis Predicting Perceived Stress and Coping Self-Efficacy

The results of the GEE analysis highlight significant differences between T_1_ and T_2_ (*p*<.01) and between T_1_ and T_3_ (*p*<.01). Furthermore, the significant difference found between the two groups in terms of mean perceived stress score (*p*<.01) revealed that participation in the yoga intervention decreased perceived stress scores by an average of 9.04 (β =−9.04, Wald 95% CI=[−7.78, −10.29]). Lastly, the significant between-group difference in coping self-efficacy scores (*p*<.01) revealed that participation in the yoga intervention increased coping self-efficacy scores by an average of 7.09 points (β=7.09, Wald 95% CI=[15.32, 1.13], *p*<.01; see Table 4).

**Table 4:** Generalized Estimating Equation Analysis Predicting Perceived Stress and Coping Self-Efficacy Scores

Item	Perceived Stress				Coping Self-Efficacy			
	*B*	*SE*	95% Wald CI	*p*	*B*	*SE*	95% Wald CI	*p*
Intercept	13.75	0.38	[13.01, 14.49]	<.01**	188.53	2.42	[183.79, 193.28]	<.01**
Group								
Intervention group versus control group	−9.04	0.64	[−7.78, −10.29]	<.01**	7.09	4.20	[15.32, 1.13]	<.01**
Time								
Posttest two versus Pretest	−1.71	0.00	[−1.71, −1.71]	<.01**	5.78	0.00	[5.78, 5.78]	<.01**
Posttest one versus Pretest	−3.33	0.00	[−3.33, −3.33]	<.01**	3.63	0.00	[3.63, 3.63]	<.01**
Interaction								
Experimental group × Posttest two	4.63	0.00	[4.63, 4.63]	<.01**	5.51	0.00	[5.51, 5.51]	<.01**
Experimental group × Posttest one	2.48	0.00	[2.48, 2.48]	<.01**	2.83	0.00	[2.83, 2.83]	<.01**

*p<.05.** *p*<.01.

## Discussion

Most of the participants in the intervention and control groups were male (*n*=23 [74%] and *n*=20 [66.7%], respectively), single (*n*=24 [77.4%] and *n*=22 [73.3%], respectively), 26–27 years old, and self-identified as homosexual (*n*=16, 51.6%; *n*=16, 53.3%). These findings are similar to those of previous studies that reported demographic information on yoga participants, showing most to be male (Quigley et al., 2020) and single (Wimberly et al., 2018). Unlike previous studies in which most participants were aged 30–50 years (Naoroibam et al., 2016; Quigley et al., 2020; Wimberly et al., 2018), the participants in this study were relatively young and identified themselves as homosexual. These differences suggest a significant change in the demographic characteristics of the HIV population in Indonesia, which is currently dominated by younger and still-productive groups. Future HIV prevention programs should place greater emphasis on younger, homosexual populations.

Also in this study, most of the participants in both groups lived alone (*n*=15 [48.4%] and *n*=18 [60%], respectively) and were socially supported by peers and friends (*n*=10 [32.3%] and *n*=8 [26.7%], respectively). These results echo those of an earlier study on 388 yoga participants conducted in India (Sakthivel & Rajendran, 2022), in which more than 50% of the participants in both groups received social support. However, the sources of social support (e.g., families, friends, peers) were not specified. Further research on this variable is needed to better elucidate the correlation between social support and psychological stress among PLWH. Moreover, our findings showed that most of the participants lived alone, suggesting a potential connection between living status and social support among PLWH. This factor may help explain why the social support received by most participants was derived mainly from friends and/or peers, rather than from family. The notable absence of the family as a source of support may relate to the rejection and stigmatization of patients with HIV (Fauk et al., 2021).

The between-group analysis results also showed a significant difference in perceived stress, with the intervention group reporting lower mean levels of stress than the control group at both T_2_ and T_3_, suggesting the intervention was effective in reducing stress and that its effects lasted through 1 month after the end of the intervention. These findings align with those of earlier investigations that demonstrated a stress reduction effect of yoga practice in HIV participants. For example, a study by Wimberly et al. (2018) conducted in Pennsylvania (USA) found that, after practicing Hatha yoga for 3 months, patients with HIV released from jail showed significantly reduced stress levels (Wimberly et al., 2018). Similarly, in a pilot study by Agarwal et al. (2015) on 22 individuals with HIV, the participants were randomly assigned to the control and yoga practice groups, with the latter receiving 2 months of yoga treatment. The intervention group reported significantly lower mean PSS scores than the control group. Furthermore, they found the stress-reducing effect of yoga to persist through 2 months after the end of the yoga intervention (Agarwal et al., 2015).

The between-group analysis in this study further identified a significant difference in coping self-efficacy between the intervention and control groups. The coping self-efficacy score had improved significantly during the eighth week of the intervention, with the effects lasting through the 12-week follow-up. This finding suggests yoga may be a highly effective tool for boosting coping self-efficacy in patients (especially those with HIV). The study by Marshall et al. (2021) on patients with chronic low back pain also supports this idea, with the authors stating that yoga participation may increase patient self-efficacy and self-motivation, which in turn may improve their mental health status, including stress. The effects of yoga on coping self-efficacy in patients have also been documented in other populations. The investigation by Hewett et al. (2018) conducted on 63 sedentary adults revealed that, after practicing 16 weeks of yoga, participants experienced greatly improved self-efficacy and significant reductions in stress. Although the positive effects of yoga on patient coping self-efficacy have been documented in many studies conducted in different populations, no evidence of these advantages has previously been identified among patients with HIV. Further research is needed to elucidate the effects of yoga on this population.

### Limitations

Biological measurements of stress, such as the cortisol test, were not used in this study. Also, the sample size was small (*n*=61), and the dropout rate was relatively high. These problems limit the ability of these findings to provide generalizable insights into the full potential of yoga interventions.

Another drawback of this RCT was its lack of external validity (applicability or generalizability). Low external validity is a problem for many RCTs due to the limitations inherent in the patient selection methods used. Thus, the generalizability of findings may be reduced. The effectiveness of this yoga intervention program, which was delivered at one clinic in one city in Indonesia only, may not necessarily represent the circumstances of all patients with HIV in Indonesia. Therefore, validation of these results using larger samples is needed. Finally, similar studies should be conducted in other Asian countries that are culturally similar to Indonesia or in different populations reflecting a greater diversity of sexual preferences.

### Conclusions

In light of the deleterious health outcomes associated with stress, there is a need to investigate the efficacy of alternative treatments (other than drugs) and convenient behavioral strategies in reducing stress in PLWH. This study adds to the existing research supporting the effectiveness of practicing yoga in reducing stress in patients with HIV. After 8 weeks of yoga practice, the mean level of stress in the intervention group had declined remarkably, while coping self-efficacy also showed improvement. Based on the results, regular yoga practice should be recommended to patients with HIV, and yoga intervention programs should be regularly provided in health care settings.

### Relevance to Clinical Practice

The yoga intervention program conducted in this study significantly and positively impacted the individuals involved, all of whom were relatively young in age. Given its success in reducing stress, this program may also benefit other populations with HIV, for example, different age groups, groups with different CD4 count levels, and patients who have only recently been infected/diagnosed with HIV. Best practice guidelines for treating PLWH suffering from stress are not yet available in Indonesia. Health care professionals may easily implement the yoga program in this study to help individuals with HIV significantly improve their stress and coping self-efficacy outcomes. The intervention program also includes yoga guidelines, complete with images and instructions for each pose. Thus, the program may help health care providers better understand yoga and facilitate its delivery to patients in need. Lastly, the results of this study may be used as guidance in health care settings and by the government and other health-promotion authorities to help patients with HIV.
